# Risk factors analysis and nomogram model construction of refeeding syndrome after esophageal cancer surgery

**DOI:** 10.3389/fonc.2026.1759898

**Published:** 2026-03-19

**Authors:** Yao Shi, Xiaoxue Liu, Yu Wang, Yun Zhou, Junjun Gu, Yanhua Li, Kexin Li, Shuying Wang, Yiqian Ni

**Affiliations:** 1 Department of Cardiovascular Surgery, The First Affiliated Hospital of Naval Medical University, Shanghai, China; 2 Department of Thoracic Surgery, The First Affiliated Hospital of Naval Medical University, Shanghai, China; 3 Nursing Department, Qingdao Traditional Chinese Medicine Hospital (Qingdao Hiser Hospital Affiliated of Qingdao University), Qingdao, China; 4 Department of Anesthesiology and Operating Theater, Shenzhen Pingle Orthopedic Hospital, Shenzhen, China

**Keywords:** esophageal cancer, logistic regression, nomogram, refeeding syndrome, risk prediction model

## Abstract

**Background:**

To construct and verify the risk prediction nomogram model of postoperative refeeding syndrome (RFS) in patients with esophageal cancer, and to provide a basis for early identification of high-risk groups and development of individualized nutritional intervention strategies.

**Methods:**

A retrospective observational study design was used. Patients who underwent esophageal cancer surgery in a Grade A tertiary hospital in Shanghai from January 2023 to August 2024 were selected as the modeling group, and patients who underwent esophageal cancer surgery from September 2024 to June 2025 were selected as the validation group. RFS was diagnosed according to the criteria of the American Society of Parenteral and Enteral Nutrition. Independent risk factors were screened by univariate analysis and multivariate logistic regression, and a nomogram model was constructed based on significant variables. Bootstrap method was used for internal validation, and independent validation group was used for external validation. The area under the receiver operating characteristic curve (AUC), Brier score, Hosmer-Lemeshow test and decision curve analysis (DCA) were used to evaluate the discrimination, calibration and clinical effectiveness of the model.

**Results:**

The total incidence of RFS was 21.74% (100/460), 22.26% (69/310) in the modeling group and 20.67% (31/150) in the validation group. Multivariate analysis showed that age, diabetes mellitus, prealbumin before feeding, albumin before feeding, parenteral nutrition support and rapid enteral feeding were independent risk factors (*P*< 0.05). Additional albumin supplementation was a protective factor (*P*< 0.05). The AUC of internal validation was 0.813 (95% CI: 0.756–0.869), and the AUC of external validation was 0.800 (95% CI: 0.709–0.890). The calibration curve fitted well, and the Hosmer-Lemeshow test *P >*0.05. DCA shows that net income can be obtained in a wide range.

**Conclusion:**

The nomogram model constructed in this study integrates core predictive factors such as age, history of diabetes, pre-feeding albumin level, nutritional support method and speed. It shows good predictive efficacy in both the internal and external validation cohorts and can provide a quantitative tool for the early screening and individualized nutritional management of patients at high risk of RFS after esophageal cancer surgery.

## 1 Introduction

Esophageal cancer is one of the common malignant tumors of the digestive tract worldwide. Radical surgery is the main treatment method. However, postoperative dysphagia, changes in gastrointestinal structure, and a hypercatabolic state place patients at a high risk of severe malnutrition. (1). Although early nutritional support can improve nitrogen balance and promote healing (2), it may also induce refeeding syndrome (RFS). RFS is an acute metabolic disorder caused by the overly rapid reintroduction of nutrition, characterized primarily by electrolyte abnormalities, especially hypophosphatemia. In severe cases, it can lead to arrhythmia and even death (3). Due to insufficient preoperative food intake, digestive and absorptive disorders, postoperative loss of digestive juices, and neoadjuvant chemoradiotherapy-induced injury, patients with esophageal cancer have significantly impaired metabolic reserve, making them an extremely high-risk group for RFS (4). The incidence of postoperative RFS in these patients ranges from 18.7% to approximately 29.3%, which not only prolongs the length of hospital stay but also significantly increases the medical burden and mortality risk (5–7).

Although RFS has received widespread attention, most existing studies have focused on patients with critical illness or chronic consumptive diseases, and systematic research targeting the specific population of patients after esophageal cancer surgery remains scarce (8, 9). Currently, there is no standardized risk assessment tool that integrates perioperative specific indicators (such as surgical methods, nutritional support rate, etc.), leading to delayed identification of high-risk patients and untimely intervention. As an intuitive quantitative tool, a nomogram can integrate multiple predictive factors to achieve individual risk stratification (10). Therefore, this study adopted a retrospective observational cohort design to systematically analyze the independent risk factors of postoperative RFS in patients with esophageal cancer, construct and validate a risk prediction nomogram, thereby providing a reliable basis for early clinical screening of high-risk patients and formulating individualized nutritional intervention strategies.

## 2 Methods

### 2.1 Study design

This study was a retrospective observational cohort study, and the subjects were patients who underwent esophageal cancer surgery and received treatment at the First Affiliated Hospital of Naval Medical University. Before the start of the study, the research purpose, research process, data collection methods, and privacy protection measures were fully informed to all enrolled patients and their families. As this study was a clinical cohort study involving human subjects, all our research procedures strictly followed the medical ethics principles related to human subjects specified in *the Declaration of Helsinki of the World Medical Association (Revised 2024)* (11), and the privacy and informed rights of the research subjects were fully protected. The research protocol was reviewed and approved by the Ethics Committee of the First Affiliated Hospital of Naval Medical University (Ethics Approval No. B2025-049), and the study was conducted in line with ethical standards without any additional research-related risks or harms to the patients.

### 2.2 Sample size calculation

Sample size was calculated according to the statistical method described in a previous study (12): $n=\frac{Z_{1-\alpha /2}^{2}\cdot P\cdot \left(1-P\right)}{d^{2}}$. According to the pre-survey, the risk of RFS in patients after esophageal cancer surgery was 20%, *d* represents the allowable error. In this study, the allowable error was controlled at 5%. Under the assumption that the confidence interval (CI) is 95%, we adopted the two-tailed test method. Considering a 20% missing rate, the required sample size is 308 cases, and finally 310 cases were included. The sample size of the risk prediction model validation group is generally 1/4 to 1/2 of the sample size of the modeling group (13). With a 10% invalid questionnaire rate, the sample size of the validation group should be at least 94 cases, and finally 150 cases were included.

### 2.3 Study subjects

The convenience sampling method was adopted. Patients with esophageal cancer who were admitted to the First Affiliated Hospital of Naval Medical University from January 2023 to August 2024 were selected as the modeling group, and patients with esophageal cancer admitted to the First Affiliated Hospital of Naval Medical University from September 2024 to June 2025 were selected as the validation group. The patients were divided into the RFS group and the non-RFS group based on whether they experienced RFS. The inclusion criteria for the patients were: (I) Patients with esophageal cancer who met the diagnostic criteria in *the Diagnosis and Treatment Guidelines for Esophageal Cancer (2022 Edition)* (14); (II) Age ≥18 years; (III) Patients who received surgical treatment; (IV) Patients with complete clinical data. The exclusion criteria were: (I) Patients with a risk of hypophosphatemia, such as those undergoing hemodialysis or those with treated hyperphosphatemia; (II) Patients undergoing parathyroidectomy; (III) Pregnant or lactating patients.

### 2.4 The judgment methods and standards of RFS

According to the definition established by the American Society for Parenteral and Enteral Nutrition (ASPEN) in 2020 (15), to determine whether a patient has RFS, it is necessary to check if there is a decrease in the concentration of one or more serum electrolytes (phosphate, potassium or magnesium), which occurs within 5 days after the start of increased energy intake. If both of the above conditions are met, it is considered as RFS. To ensure the accuracy of the judgment, within 5 days after the patient receives enteral nutrition support, the attending physician and the responsible nurse jointly determine whether it is RFS.

### 2.5 Study tools

#### 2.5.1 General information questionnaire

Based on the results of literature search and expert consultation, the research team initially selected 26 potential influencing variables (1). Basic demographic information: including body mass index (BMI), age, gender, smoking history, drinking history, etc. (2); Disease data: surgical history, underlying diseases, whether insulin, diuretics, acid suppressants and antibiotics were used before feeding, etc. Among them, having a smoking history was defined as smoking more than 1 cigarette per day for more than 1 year; having a drinking history was defined as drinking at least 5 days per week and with a drinking volume of more than 50ml per day for more than 6 months.

#### 2.5.2 Nutritional risk screening scale

The nutritional risk screening scale (NRS) was developed and recommended by the European Society of Clinical Nutrition and Metabolism (16), and it consists of three dimensions: age, nutritional status, and physical condition. Among them, a score of 1 is given for those aged 70 or above, and 0 for those under 70; a score of 0 is given for normal nutritional status, 1 point is given if the weight has decreased by more than 5% within 3 months or if the food intake within 1 week is 50% to 75% of the normal requirement, 2 points if the weight has decreased by more than 5% within 2 months and BMI is between 18.5 and 20.5 or if the food intake within 1 week is 25% to 50% of the normal requirement, and 3 points if the weight has decreased by more than 5% within 1 month and BMI is less than 18.5 or if the food intake is less than 25% of the normal requirement within 1 week; a score of 0 is given for normal physical condition, 1 point is given if the physical condition is weak but still able to get out of bed regularly, 2 points if long-term bed rest is required, and 3 points if the disease is severe and the acute physiology and chronic health status II (APACHE II) score is greater than 10; the total NRS score ranges from 0 to 7, with a higher score indicating a higher nutritional risk for the patient. The Cronbach’s α coefficient of this scale is 0.670.

#### 2.5.3 Acute physiology and chronic health evaluation scale

The APACHE II score was revised by Knaus et al. (17) in 1985. It consists of three components: acute physiological indicators (body temperature, blood pressure, heart rate, respiratory rate, blood oxygen partial pressure, arterial blood pH value, sodium concentration, etc.), age, and chronic health status. The score ranges from 0 to 6 points for the patient’s condition, 0 to 21 points, and 0 to 44 points respectively, with a total score ranging from 0 to 71 points. The higher the score, the more severe the patient’s condition. The Cronbach’s α coefficient of this scale is 0.790.

### 2.6 Data collection method

Two nurses who have received unified training collected and organized the general information, disease information, re-feeding-related information, NRS scores, and APACHE II scores of the patients through the hospital’s electronic medical record system and nursing records. They also conducted a double-entry verification to check for errors and omissions. Any incorrect or missing items were promptly filled in to ensure the accuracy of the entered data.

### 2.7 Statistical methods

The data were processed using SPSS 26.0 statistical software. All the measurement data were normal and were expressed as mean ± standard deviation. The comparison between groups was conducted using the two independent samples t-test. The count data were expressed as the number of cases, percentages or rates, and the comparison between groups was performed using the χ^2^ test, rank sum test. With esophageal cancer patients’ occurrence of RFS as the dependent variable, the factors with statistical significance in the univariate analysis were used as independent variables to conduct Logistic regression analysis to identify the independent risk factors. The risk prediction model nomogram, the area under the receiver operating characteristic curve (AUC), and calibration curve were drawn using Rstudio 4.4.3 software. The goodness of fit of the model was evaluated using the Hosmer-Lemeshow goodness-of-fit test and calibration curve, and internal and external validations were conducted. A difference was considered statistically significant if *P*< 0.05.

## 3 Results

### 3.1 Patient general information

**Figure 1:**
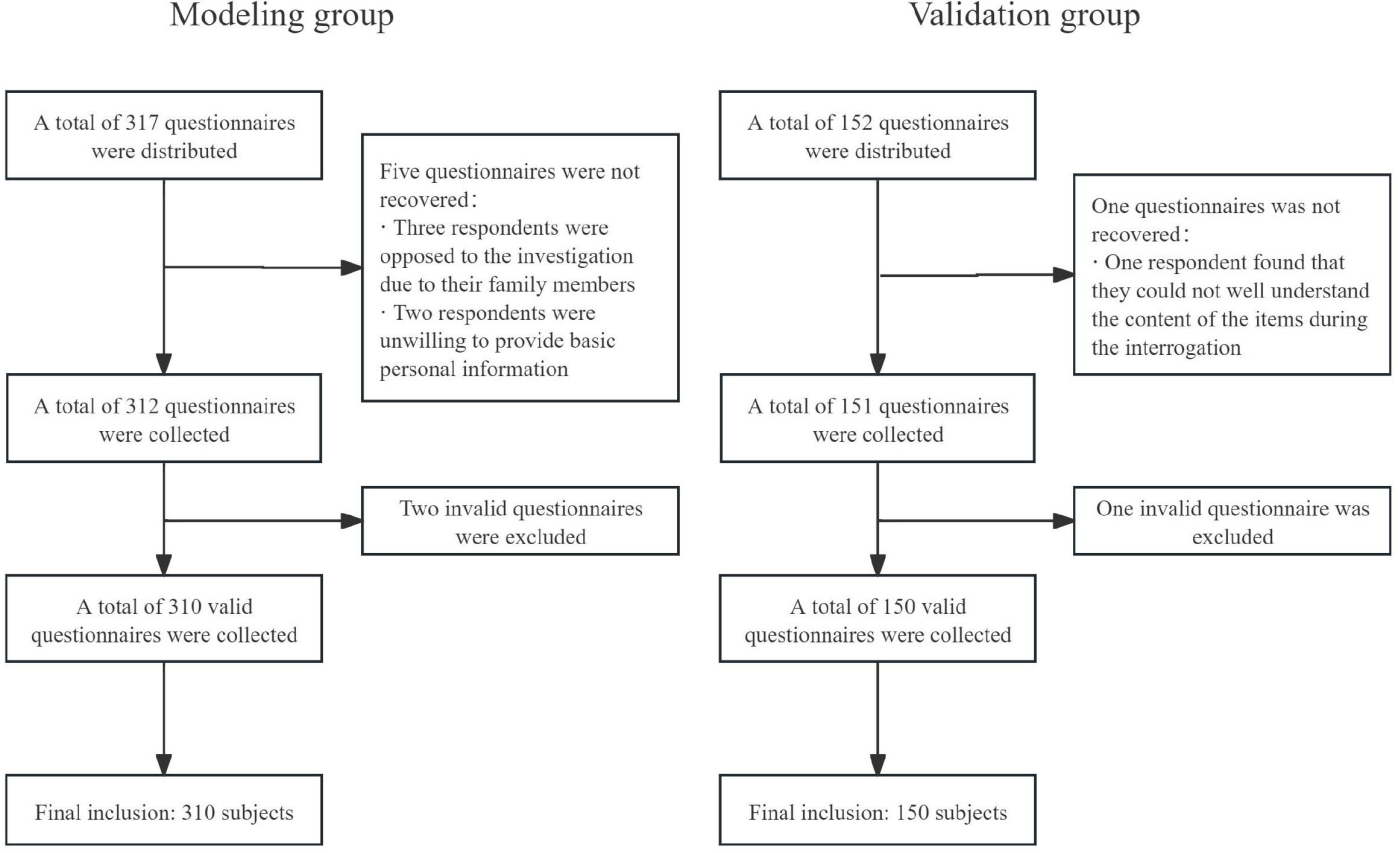
Flowchart of the inclusion process for the modeling group and the validation group study subjects.

This study ultimately included 460 patients with esophageal cancer, and the RFS rate was 21.74% (100/460). In the modeling group, the RFS accounted for 22.26% (69/310); in the validation group, the RFS accounted for 20.67% (31/150). Details can be found in **Figure 1**. Among them, male patients accounted for 61.96% (285/460), and female patients accounted for 38.04% (175/460).

### 3.2 Single-factor analysis results of RFS in patients with esophageal cancer after surgery

The results of the single-factor analysis showed that there were statistically significant differences between the two groups in terms of age, BMI, presence of diabetes, pre-feeding prealbumin, prealbumin before feeding, albumin before feeding, feeding rate, additional albumin supplementation, APACHE II score, operation time, and tumor node metastasis (TNM) staging (*P*< 0.05), as shown in **Table 1**.

**Table 1:** Results of the single analysis of risk factors for RFS in patients with esophageal cancer (n=310).

Factors	Level	Non-RFS group (241)	RFS group (69)	*χ* ^2^	*P* value
Gender (%)	Man	148 (61.41)	46 (66.67)	0.633	0.426
	Woman	93 (38.59)	23 (33.33)		
Age (%)	<60	82 (34.02)	12 (17.40)	7.025	0.008
	≥60	159 (65.98)	57 (82.61)		
BMI (%)	Underweight	14 (5.81)	11 (15.94)	7.545	0.023
	Normal	155 (64.32)	41 (59.42)		
	Overweight	72 (29.86)	17 (24.64)		
Smoking history (%)	Yes	103 (42.74)	35(50.72)	1.385	0.239
	No	138 (57.26)	34 (49.28)		
Alcohol consumption history (%)	Yes	93 (38.59)	28 (40.58)	0.089	0.765
	No	148 (61.41)	41 (59.42)		
Previous surgical history (%)	Yes	69 (28.63)	17 (24.64)	0.427	0.514
	No	172 (71.37)	52 (75.36)		
Diabetes (%)	Yes	26 (10.79)	18 (26.09)	10.308	0.001
	No	215 (89.21)	51 (73.91)		
Hypertension (%)	Yes	87 (36.10)	21 (30.43)	0.758	0.384
	No	154 (63.90)	48 (69.57)		
Prealbumin before feeding (%)	<150	32 (13.28)	20 (28.99)	9.480	0.002
	≥150	209 (86.72)	49 (71.01)		
Albumin before feeding (%)	<30	44 (18.26)	23 (33.33)	7.197	0.007
	≥30	197 (81.74)	46 (66.77)		
Pre-feeding fasting period (%)	<2d	150 (62.24)	37 (53.62)	1.664	0.197
	≥2d	91 (37.76)	32 (46.38)		
Parenteral nutrition support (%)	Yes	189 (78.42)	39 (56.52)	13.266	0.000
	No	52 (21.58)	30 (43.48)		
Feeding rate (%)	20 ml/h	38 (15.77)	5 (7.25)	11.685	0.003
	20–50 ml/h	138 (57.26)	31 (44.93)		
	>50 ml/h	65 (26.97)	33 (47.83)		
Type of nutrient solution (%)	Whole protein	135 (56.02)	33(47.83)	1.450	0.229
	Short peptide	106 (43.98)	36 (52.17)		
Additional albumin supplementation (%)	Yes	112 (46.47)	21 (30.43)	5.633	0.018
	No	129 (53.53)	48 (69.57)		
Heated infusion (%)	Yes	118 (48.96)	31 (44.93)	0.350	0.554
	No	123 (51.04)	38 (55.07)		
Continuous infusion time (%)	<12 h/d	138 (57.26)	44 (63.77)	0.937	0.333
	≥12 h/d	103 (42.74)	25 (36.23)		
NRS2002 (%)	<3 points	119 (49.38)	29 (42.03)	1.161	0.281
	≥3 points	122 (50.62)	40 (57.97)		
APACHE II Score (%)	<20 points	205 (85.06)	51 (73.91)	4.635	0.031
	≥20 points	36 (14.94)	18 (26.09)		
Operation time (%)	<5h	138 (57.26)	34 (49.28)	136.468	0.000
	≥5h	103 (42.74)	35 (50.72)		
Preoperative chemotherapy (%)	Yes	31 (12.86)	7 (10.14)	0.368	0.544
	No	210 (87.14)	62 (89.86)		
TNM staging (%)	I–II	116 (48.13)	29 (42.03)	16.674	0.000
	III	118 (48.96)	29 (42.03)		
	IV	7 (2.90)	11 (15.94)		
Tumor location (%)	The upper segment	41 (17.01)	12 (17.40)	0.092	0.955
	Midsection	122 (50.62)	36 (52.17)		
	The lower segment	78 (32.37)	21 (30.43)		
Histological type (%)	Squamous cell carcinoma	172 (71.37)	50 (71.46)	1.272	0.529
	Adenocarcinoma	46 (19.09)	10 (14.50)		
	Others	23 (9.54)	9 (13.04)		

### 3.3 Multivariate analysis results of RFS in patients with esophageal cancer after surgery

Taking whether esophageal cancer patients experienced RFS as the dependent variable and the items with statistically significant differences in the univariate analysis as the independent variables, a Logistic regression analysis was conducted. The age, BMI, diabetes mellitus, prealbumin before feeding, albumin before feeding, parenteral nutrition support, feeding rate, additional albumin supplementation, APACHE II score, operation time, and TNM staging assignment method are shown in **Table 2**. The results of the Logistic regression analysis indicated that age, diabetes mellitus, prealbumin before feeding, albumin before feeding, parenteral nutrition support, feeding rate were independent risk factors for esophageal cancer patients experiencing RFS, and additional albumin supplementation was a protective factor for esophageal cancer patients experiencing RFS (*P*< 0.05), as shown in **Table 3**.

**Table 2:** Assignment method of independent variables.

Independent variable	Assignment method
Age	<60 years old = 0, ≥60 years old = 1
BMI	Normal (18.5–24.0) = 1, Underweight (<18.5) = 2, Overweight (>24.0) = 3
Prealbumin before feeding	≥150 mg/L = 1,<150 mg/L = 2
Albumin before feeding	≥30 g/L = 1,<30 g/L = 2
Parenteral nutrition support	No = 1, Yes^1^ = 2
Feeding rate	20 ml/h = 1, 20–50 ml/h = 2, >50 ml/h = 3
Additional albumin supplementation	No = 0, Yes^2^ = 1
APACHE II score	<20 points = 0, ≥20 points = 1
Operation time	<5h = 1, ≥5h = 2
TNM staging	Stages I–II = 1, Stages III = 2, Stages IV = 3

1, Receiving parenteral nutrition support after surgery; 2, Additional intravenous supplementation of albumin during the perioperative period.

**Table 3:** Logistic regression analysis results of risk factors for refeeding syndrome in patients with esophageal cancer (n=310).

Independent variable		β value	SE value	Wald χ^2^ value	*P* value	OR value	95% CI
Constant term		-7.971	1.206	43.681	0.000	Not mentioned	Not mentioned
Age		1.051	0.396	7.048	0.008	2.861	1.317–6.216
Diabetes		1.329	0.419	10.057	0.002	3.779	1.662–8.594
Prealbumin before feeding		1.147	0.525	4.765	0.029	3.148	1.124–8.813
Albumin before feeding		1.803	0.675	7.125	0.008	6.066	1.614–22.794
Parenteral nutrition support		1.293	0.338	14.652	<0.001	3.643	1.879–7.063
Feeding rate	<20 ml/h	Not mentioned	Not mentioned	14.787	<0.001	Not mentioned	Not mentioned
	20–50 ml/h	0.791	0.567	1.945	0.163	2.206	0.726–6.705
	>50 ml/h	1.845	0.580	10.125	0.001	6.328	2.031–19.715
Additional albumin supplementation		-2.161	0.577	14.036	<0.001	0.115	0.037–0.357

### 3.4 Construction of a RFS nomogram model for esophageal cancer patients after surgery

**Figure 2:**
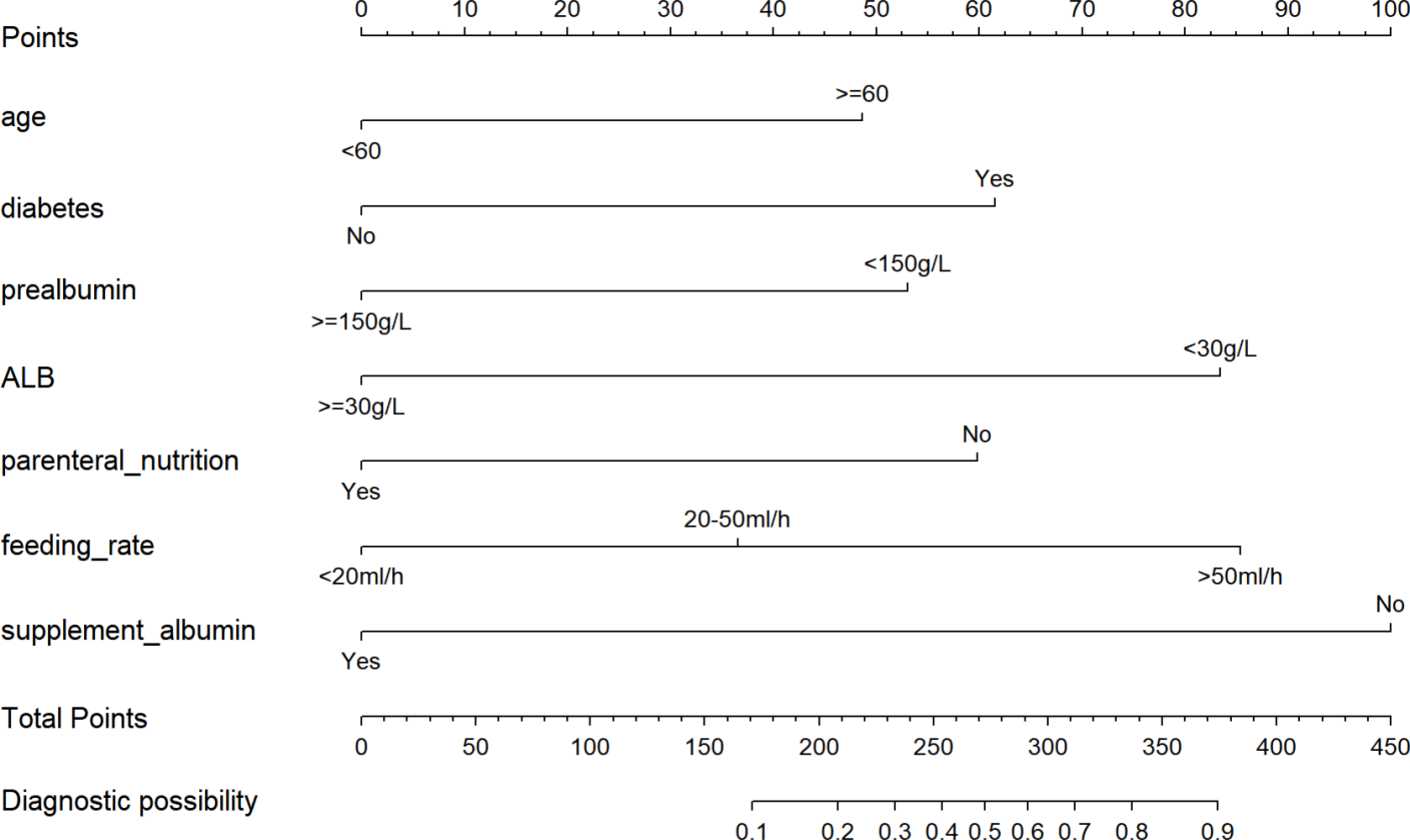
Nomogram for risk prediction of RFS after esophageal cancer surgery. RFS, refeeding syndrome. A nomogram model was developed to estimate the risk of refeeding syndrome in patients after esophageal cancer surgery. The total points were calculated as the sum of the points assigned to each predictor, and the corresponding predicted probability was obtained from the bottom scale of the nomogram. For example, a total score of 270 indicated a probability of more than 50%, while a total score of 375 indicated a probability of more than 90%.

A nomogram model for predicting the occurrence of RFS in esophageal cancer patients was established based on age, coexisting diabetes, prealbumin before feeding, albumin before feeding, parenteral nutrition support, feeding rate, and additional albumin supplementation, as shown in **Figure 2**. The application method of the nomogram model: Draw a perpendicular line downward from the corresponding segment based on the RFS variable conditions of esophageal cancer patients, obtain the corresponding score value for each variable, calculate the risk score for each predictive factor according to this principle, and add up the scores of all factors to find the corresponding predictive probability, thereby obtaining the risk probability of RFS occurrence in esophageal cancer patients.

### 3.5 Validation of the postoperative RFS nomogram model for esophageal cancer patients

The predictive efficacy of the model was evaluated from three aspects: discrimination, calibration, and clinical effectiveness. Discrimination was assessed using AUC, calibration was evaluated using Brier score, Hosmer-Lemeshow test, and calibration curve graph, and clinical effectiveness was evaluated using DCA.

**Figure 3:**
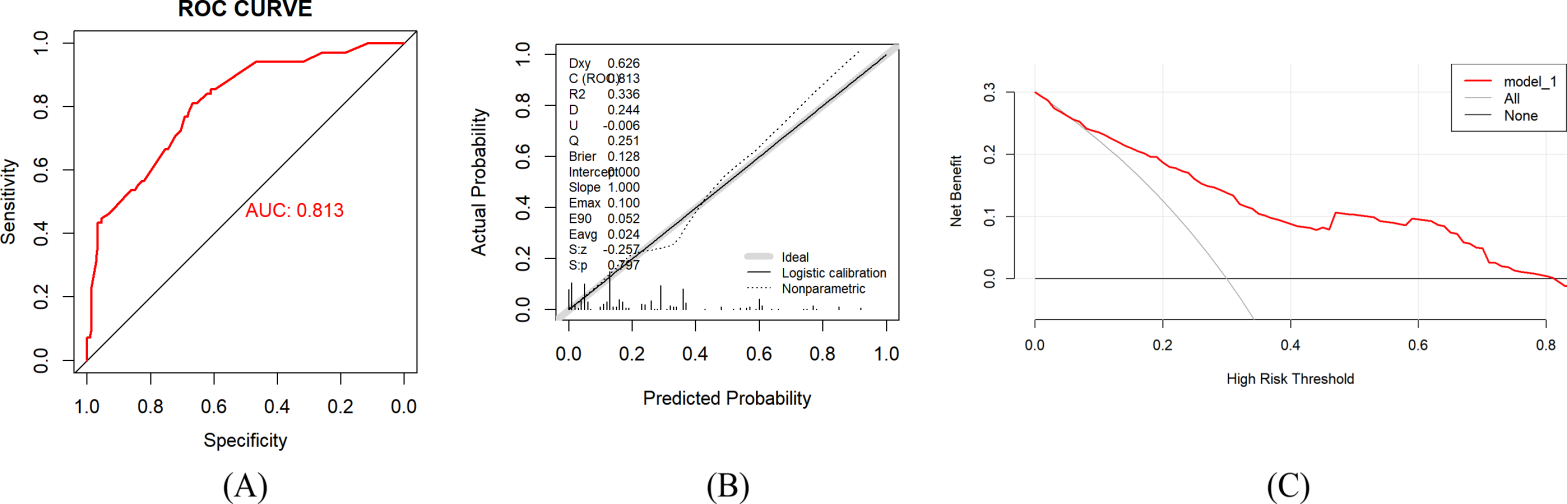
**(A)** ROC curve of the modeling group. AUC = 0.813 (95% CI: 0.756–0.869). **(B)** Calibration curve of the training group. The calibration curve was used to evaluate the consistency between the predicted probabilities and the observed outcomes, with the diagonal line representing perfect calibration. The Hosmer-Lemeshow goodness-of-fit test showed no significant deviation between the predicted risk and the observed risk (*P* = 0.365), indicating that the model had good calibration. **(C)** DCA curve of the modeling group. The vertical axis represents the net benefit, and the horizontal axis represents the threshold probability. This curve compares the “treat all” strategy and the “treat none” strategy; the higher the net benefit of the former, the better the clinical utility. In the figure, the light gray curve corresponds to the “treat all” strategy, and the black curve corresponds to the “treat none” strategy.

The internal validation of the modeling data was conducted by repeating the Bootstrap sampling 1000 times. The AUC value was 0.813 (95% CI: 0.756–0.869), as shown in **Figure 3A**. The Brier score was 0.128 (95% CI: 0.105–0.151). The calibration curve results indicated that the predicted RFS probability of esophageal cancer patients by the model was highly consistent with the actual RFS probability, as shown in **Figure 3B**. The Hosmer-Lemeshow test χ^2^ value was 9.820, *P* = 0.365, indicating that there was no statistical difference between the predicted risk and the actual discovered risk of the model. In addition, the decision curve results showed that within the probability threshold range of 0.10 to 0.80, using this model for clinical decision-making could achieve a greater net benefit compared to the “no intervention” or “full intervention” schemes, as shown in **Figure 3C**.

**Figure 4:**
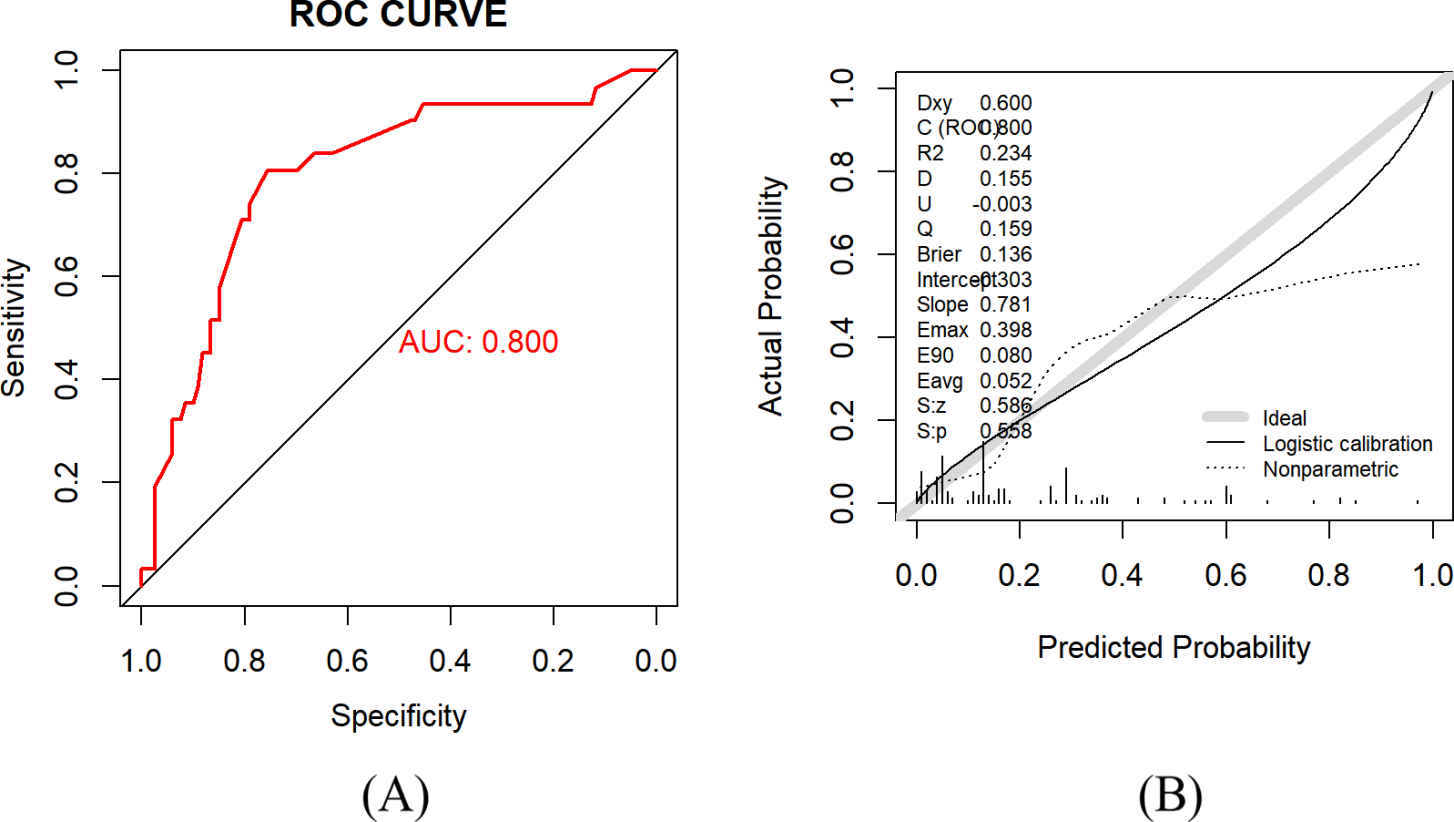
**(A)** ROC curve of the validation group. AUC = 0.800 (95% CI: 0.709–0.890). **(B)** Calibration curve of the validation group. The calibration curve was used to evaluate the consistency between the predicted probabilities and the observed outcomes, with the diagonal line representing perfect calibration. The Hosmer-Lemeshow goodness-of-fit test showed no significant deviation between the predicted risk and the observed risk (*P* = 0.316), indicating that the model had good calibration.

The model was externally validated using the validation group, with an AUC value of 0.800 (95% CI: 0.709–0.890), as shown in **Figure 4A**, indicating that the model still has good discrimination in the external validation. The Brier score was 0.136 (95% CI: 0.101–0.171). The calibration curve results showed that the RFS prediction probability of the model was in good agreement with the actual probability in the validation group, as shown in **Figure 4B**. The Hosmer-Lemeshow test χ^2^ value = 10.437, *P* = 0.316, indicating that the fit degree between the expected probability and the actual occurrence probability in the validation group of the model was good.

## 4 Discussion

Among the 460 patients with esophageal cancer who underwent surgery included in this study, the overall incidence of RFS was 21.74% (100/460), which was highly consistent with the 18.7%–29.3% reported in the literature (5–7), further confirming the high incidence of this complication in the population of patients after esophageal cancer surgery. Multivariate Logistic regression analysis identified 6 independent risk factors (advanced age ≥60 years, complicated with diabetes mellitus, prealbumin before feeding<150 mg/L, hypoalbuminemia<30 g/L, parenteral nutritional support, rapid enteral feeding >50 ml/h) and 1 protective factor (perioperative additional albumin supplementation). Their association mechanisms were closely related to the metabolic characteristics of patients with esophageal cancer: hypoalbuminemia and low prealbumin represent chronic malnutrition and acute catabolism, respectively, which often impair tissue repair capacity and amplify insulin-mediated electrolyte influx (18, 19); diabetes mellitus exacerbates electrolyte disorders through insulin resistance, and elderly patients have reduced tolerance to electrolyte fluctuations due to decreased renal function (20, 21); the high glucose load of parenteral nutrition and intestinal mucosal injury caused by rapid enteral feeding further deplete electrolyte reserves (22); while albumin supplementation may exert a protective effect by maintaining colloid osmotic pressure and buffering phosphorus transfer (23).

The nomogram risk prediction model constructed in this study has significant innovation: first, it innovatively integrates specific indicators of RFS after esophageal cancer surgery, including nutritional support operation parameters (enteral feeding rate and parenteral nutrition use) and metabolic indicators (prealbumin before feeding, albumin before feeding), which effectively makes up for the deficiency of traditional assessment tools in paying attention to postoperative dynamic indicators (24) and solves the problem of delayed early warning of general assessment tools; second, the model incorporates perioperative albumin supplementation as a protective factor, providing a clear target for clinical intervention, while most existing similar studies only focus on risk factors and lack the integration of protective factors (10, 25); third, the AUC of internal validation was 0.813 (95% CI: 0.756–0.869), and the AUC of external validation was 0.800 (95% CI: 0.709–0.890), confirming that the model has good discrimination and calibration. DCA showed that significant net benefits could be obtained within the probability threshold range of 0.10–0.80, and its quantitative efficacy was superior to that of existing non-specific prediction models (8, 9).

The clinical value of this model is mainly reflected in three aspects: first, efficient risk stratification: clinicians can quickly calculate the probability of RFS occurrence in patients through the nomogram, and initiate intensive monitoring for high-risk populations with advanced age, diabetes mellitus, and hypoalbuminemia; second, guiding individualized nutritional intervention: nurses can adjust feeding strategies according to risk levels; for high-risk patients, a low initial rate (10–30 ml/h) and stepwise increment should be adopted, and albumin levels should be dynamically monitored with targeted supplementation to avoid metabolic risks associated with rapid feeding and parenteral nutrition (22); third, optimizing the allocation of medical resources: enhanced dynamic monitoring of electrolytes should be performed in medium and high-risk patients 3–7 days after surgery, while the monitoring interval can be prolonged in low-risk patients. At the same time, targeted health education should be carried out for high-risk groups such as the elderly and patients with diabetes mellitus to improve treatment compliance (25). The multidisciplinary team collaboration, where clinicians formulate plans, nurses monitor operations, and the nutrition team adjusts strategies, forms a closed-loop management, which helps reduce the incidence of RFS after esophageal cancer surgery.

This study has the following limitations: first, convenience sampling from a single center was adopted, and both the modeling group and the validation group were from the same medical center. The representativeness of the sample may be limited, and caution should be exercised when extrapolating the conclusions to medical institutions in different regions and at different levels. Second, this study was a retrospective observational cohort study focusing on the screening of clinical risk factors and the construction of prediction models. No *in vivo* and *in vitro* experiments were conducted to explore the molecular mechanism of RFS occurrence, so the causal relationship and regulatory pathways between predictors and RFS cannot be clarified. Third, there are certain limitations in the selection of predictive variables: surgical-related specific indicators, inflammatory markers, and dynamic changes of metabolic indicators were not integrated, which may affect the predictive efficacy of the model. Finally, the current model was constructed using the traditional Logistic regression algorithm, so there is still room for improvement in prediction accuracy. In addition, it lacks a dynamic online prediction tool, resulting in insufficient clinical accessibility.

In response to the above limitations, future studies will focus on the following work: ① conduct multi-center collaborative studies, expand the sample size, and include patients from medical institutions in different regions and at different levels to improve the external applicability of the model; ② enrich the dimension of predictive variables, integrate data such as surgical details, inflammatory markers, and dynamic monitoring of metabolic indicators to optimize the model structure; ③ explore machine learning algorithms to further improve the prediction accuracy and stability of the model; ④ develop a dynamic online prediction platform to simplify the risk assessment process and enhance the convenience of clinical application; ⑤ conduct *in vivo* and *in vitro* experiments to deeply explore the molecular mechanism of RFS, clarify the regulatory role of key predictors, and provide more accurate targets for clinical intervention.

## 5 Conclusion

This study conducted a multivariate Logistic regression analysis to systematically identify the core independent risk factors for postoperative RFS of esophageal cancer: advanced age, diabetes mellitus, prealbumin before feeding and albumin before feeding, parenteral nutrition support, and rapid enteral feeding. At the same time, the protective effect of perioperative albumin supplementation was clarified. Based on these factors, a nomogram prediction model was constructed, which integrated metabolic indicators, nutritional operation parameters, and the patient’s metabolic background, effectively compensating for the lack of attention to postoperative dynamic indicators by traditional tools. The model was confirmed to have excellent discrimination through internal and external validation, and the calibration curve and decision analysis further verified its clinical applicability. This model can quickly identify high-risk patients through visualization tools, guide nursing staff to implement protective feeding strategies, and conduct differentiated monitoring of electrolytes based on risk stratification, providing an important practical tool for reducing the incidence of metabolic crises after esophageal cancer surgery. In the future, the model will be continuously improved through multicenter collaboration, enrichment of predictive variables, algorithm optimization, and development of an online platform. Combined with *in vivo* and *in vitro* experiments, we will further explore the mechanism underlying RFS, so as to provide more comprehensive theoretical and practical support for the precise prevention and control of RFS after esophageal cancer surgery.

## Data Availability

The original contributions presented in the study are included in the article/supplementary material. Further inquiries can be directed to the corresponding author.
